# CK2 Secreted by *Leishmania braziliensis* Mediates Macrophage Association Invasion: A Comparative Study between Virulent and Avirulent Promastigotes

**DOI:** 10.1155/2015/167323

**Published:** 2015-05-18

**Authors:** Ana Madeira Brito Zylbersztejn, Carlos Gustavo Vieira de Morais, Ana Karina Castro Lima, Joyce Eliza de Oliveira Souza, Angela Hampshire Lopes, Sílvia Amaral Gonçalves Da-Silva, Mário Alberto Cardoso Silva-Neto, Patrícia Maria Lourenço Dutra

**Affiliations:** ^1^Laboratório de Bioquímica de Protozoários e Imunofisiologia do Exercício, Disciplina de Parasitologia, DMIP, FCM, Universidade do Estado do Rio de Janeiro, Rua Professor Manuel de Abreu, 444, Pavilhão Américo Piquet Carneiro, 5° Andar, 20550-170 Rio de Janeiro, RJ, Brazil; ^2^Programa de Pós Graduação em Microbiologia/FCM/UERJ, Av. Professor Manuel de Abreu, 444, Pavilhão Américo Piquet Carneiro, 3° andar, Vila Isabel, 20550-170, Rio de Janeiro, RJ, Brazil; ^3^Instituto de Microbiologia Paulo de Góes-CCS, Universidade Federal do Rio de Janeiro (UFRJ), Avenida Carlos Chagas Filho, 373, CCS, Bloco I, Ilha do Fundão, 21941-590 Rio de Janeiro, RJ, Brazil; ^4^Laboratório de Imunofarmacologia Parasitária, Disciplina de Parasitologia, DMIP, FCM, Universidade do Estado do Rio de Janeiro, Rua Professor Manuel de Abreu 444, Pavilhão Américo Piquet Carneiro, 5° Andar, 20550-170 Rio de Janeiro, RJ, Brazil; ^5^Laboratório de Sinalização Celular, Instituto de Bioquímica Médica-CCS, Universidade Federal do Rio de Janeiro (UFRJ), Avenida Carlos Chagas Filho, 373, CCS, Bloco H, Ilha do Fundão, 21941-590 Rio de Janeiro, RJ, Brazil

## Abstract

CK2 is a protein kinase distributed in different compartments of *Leishmania braziliensis:* an externally oriented ecto-CK2, an intracellular CK2, and a secreted CK2. This latter form is constitutively secreted from the parasite (CsCK2), but such secretion may be highly enhanced by the association of specific molecules, including enzyme substrates, which lead to a higher enzymatic activity, called inductively secreted CK2 (IsCK2). Here, we examined the influence of secreted CK2 (sCK2) activity on the infectivity of a virulent *L. braziliensis* strain. The virulent strain presented 121-fold higher total CK2 activity than those found in an avirulent strain. The use of specific CK2 inhibitors (TBB, DRB, or heparin) inhibited virulent parasite growth, whereas no effect was observed in the avirulent parasites. When these inhibitors were added to the interaction assays between the virulent *L. braziliensis* strain and macrophages, association index was drastically inhibited. Polyamines enhanced sCK2 activity and increased the association index between parasites and macrophages. Finally, sCK2 and the supernatant of the virulent strain increased the association index between the avirulent strain and macrophages, which was inhibited by TBB. Thus, the kinase enzyme CK2 seems to be important to invasion mechanisms of *L. braziliensis*.

## 1. Introduction

*Leishmania braziliensis* is an etiological agent of leishmaniasis in the New World [1] that can differentiate from avirulent to virulent promastigotes in the sandfly midgut and from promastigotes to amastigotes in mammalian macrophages. The relationship between the parasite and host cells commonly involves signal transduction pathways that are triggered upon the interaction between the surfaces of both parasites and macrophages [2].

The invasion of host immune cells by pathogens is the first step of a complex series of events that ultimately allow parasite proliferation and infection of a whole organism. The presence of enzyme activities on the parasite surface can promote the subversion of host cell membrane proteins that regulate access to the intracellular environment. Once inside, the parasite must ensure their replication and the suppression of host immunity. This process involve complex signal transduction pathways that use the reversible phosphorylation of proteins promoted by protein phosphatases and kinases, which continuously modulate the host-pathogen interaction [3]. In this manner, active enzyme secretion following invasion is likely associated with host cell machinery subversion. Evidence for the existence of such a mechanism is available in several models of host and pathogen interaction [4].

Protein kinases are involved in many cellular processes [5]. This family has hundreds of members capable of phosphorylating specific amino acid residues, such as serine, threonine, or tyrosine, as its target proteins [6–8].

Two families of protein casein kinase (CK), CK1 and CK2, whose name is due to the fact that casein is a suitable substrate, are commonly used for* in vitro* activity testing. Unlike CK2, CK1 protein kinase is used as the only phosphate ATP donor [9].

*L. braziliensis* genome analysis by TriTrypDB has shown that the ck2 gene has a single copy, located on chromosome 34.

CK2 protein kinase frequently appears as a quaternary structure, with a molecular weight of approximately 130 kDa, composed of two types of catalytic (*α*) and regulatory (*β*) subunits, and a general structure of *αα*′*β*2 or *α*2*β*2 [8–10]. The *α* and *α*′ subunits are catalytically active and have molecular weights between 42–44 kDa or 38 kDa, respectively. They are structurally similar but encoded by different genes [9, 11, 12] and have distinct functions [8]. A third subunit isoform, designated as *α*′′, also occurs [13, 14]. The *β* regulatory subunit (approximately 26 kDa in animal cells) is naturally inactive but enhances the catalytic activity of *α*5-10-fold [9] and can interact with other proteins [15]. The association between the *α* and *β* subunits determines the sphere protein structure. The complex is stabilized by the C-terminal domain, which is involved in the strong interaction between the two subunits through four electrostatic interactions between the positively charged region of the catalytic site (*α*) and a highly negative region in the regulatory subunit (*β*). This closed configuration is responsible for the catalytic site of an obstruction, which allows restricted access to protein substrates [16]. In this manner, positively charged molecules are able to modulate CK2 activity to promote the exposure of the active enzyme site. Thus, negative molecules (such as heparin) inhibit this activity, whereas positive molecules (such as polyamines) increase this activity [8]. In the absence of negative charges, CK2 maintains a stable and closed conformation. In contrast, in the presence of positive compounds, the *β* subunit is dislocated allowing the enzyme to assume the open conformation less stable, which facilitates substrate access [16].

This protein is highly conserved in nature [17] and regulates cell development and differentiation and the cell cycle [18]. This enzyme is inhibited by heparin [9] and by cell-permeable drugs, such as DRB (5,6-dichlorobenzimidazone-1-*β*-D-ribofuranoside) [19] and TBB (4,5,6,7-tetrabromobenzotriazole) [20]. CK2 can be found in the nucleus, in the cytoplasm, and on the cellular surface of mammalian cells [18] and has been previously described on the surface of intact cells [21]. Its secretion may be induced by its potential phosphorylatable substrates, such as casein and phosvitin [22, 23]. The presence of CK2 activity has been previously described in trypanosomatids [23–26]. Moreover, when these organisms were incubated in the dephosphorylated casein presence, increased CK2 secretion was observed [23, 26].

Some papers have described CK2 activity in various protozoan parasites species, such as* L. tropica* [23],* L. major *[25],* L. amazonensis* [24],* L. donovani* [27],* Herpetomonas muscarum muscarum *[26],* Trypanosoma cruzi* [28],* T. brucei *[29], and* Toxoplasma gondii* [30]. Furthermore, constitutive and inducible CK1 and CK2 activities are present on the surface of cells and in enzymatic secretions from* L. major* promastigotes; therefore, these proteins appear to be involved in* L. major* and* L. amazonensis* promastigote cell growth, morphology, and infectivity in* in vitro* mice peritoneal macrophages and* in vivo* BALB/c mice [25]. The participation of these enzymes in the parasite-host interaction may be crucial for successful infection.

In the present study, we identified and characterised secreted CK2 (sCK2) enzyme activity in supernatants of highly virulent* L. braziliensis* promastigotes. In addition, we evaluated the contribution of this enzyme in parasite survival and infectivity in host cells.

## 2. Material and Methods

### 2.1. Microorganisms and Growth Conditions

We used promastigotes of* L. braziliensis* virulent strain (MHOM/BR/2002/EMM IOC-L2538) cultured for 60 passages until it became avirulent. This strain was kindly provided by Dr. Léa Cysne. The promastigotes were grown in Schneider's medium supplemented with 10% foetal calf serum at 28°C. The amastigotes were isolated from the lymph nodes of hamsters infected 8 to 10 weeks earlier. The parasites were maintained in culture (*in vitro*) for no more than 8 passages to maintain infectivity. Six days after culture* in vitro*, the parasites were harvested by centrifugation, washed twice with 0.9% saline, and washed once with a buffer containing 20 mM Tris-HCl, pH 7.5, 10 mM NaF, 1 mM sodium orthovanadate, 150 mM NaCl, 1 mM glucose, and 1 mM MgCl_2_. Then, the parasites were maintained in the same buffer until the protein kinase activity assays were performed. Cell viability was assessed before and after incubation by motility and cell dye exclusion (Trypan blue method) [31]. The viability of the parasites was not affected by our laboratory conditions or procedures. For the experiments on cellular interaction, the parasites were washed in RPMI 1640 medium and maintained in this medium until the experiments were performed.

### 2.2. Growth Course

The parasites (virulent and avirulent promastigotes) were grown in Schneider's medium supplemented with 10% foetal calf serum at 28°C in the absence or presence of heparin (10 *µ*g/mL), TBB (1 *µ*M), DRB (6 *µ*M), and casein (1 mg/mL) for 6 days. The number of cells was evaluated after each 24 h period using Neubauer's chamber.

### 2.3. Induction of sCK2 Secretion

Intact promastigotes were incubated in a buffer containing 20 mM Tris-HCl, pH 7.5, 150 mM NaCl, 10 mM NaF, 1 mM sodium orthovanadate, 1 mM glucose, and 1 mM MgCl_2_ in the absence or presence of dephosphorylated casein (1 mg/mL), albumin (1 mg/mL), foetal bovine serum (1 mg/mL), inactivated human serum (1 mg/mL), and fixed BALB/c mice peritoneal macrophages for 30 minutes at 37°C. The parasites were centrifuged, and the supernatant was analysed to determine CK2 activity as follows. To assess the molecular mass of sCK2, intact promastigotes were incubated in the presence of dephosphorylated casein (1 mg/mL) for 30 min at 37°C. Next, the cells were centrifuged to obtain the supernatant, which was applied to a gel filtration superose 6H/R column coupled to a Shimadzu HPLC system as previously described [26]. All harvested fractions were assayed for CK2 activity [32] using TBB (1 *µ*M) and heparin (10 *µ*g/mL) as inhibitors to identify the fraction of enzyme activity corresponding to CK2.

### 2.4. Protein Kinase Activity

Protein kinase activities from intact promastigotes (ecto-CK2, eCK2), cytoplasmic contents (iCK2), and the supernatants of parasites medium of incubation (sCK2) were measured as previously described [32]. iCK2 was obtained by three cycles of parasite freeze and thaw, followed by centrifugation. Each of the above mentioned CK2s was assayed as follows. Briefly, either dephosphorylated casein (5 mg/mL) or the CK2-specific peptide RRRADDSDDDDD (50 *µ*M) [23, 27] was used as substrate in the presence of 100 *µ*M ATP-[*γ*-^32^P] (1,000 cpm/pmol) in the following reaction mixture: 20 mM Tris-HCl, pH 8.0, 150 mM NaCl, 1 mM EDTA, 1 mM EGTA, 1 mM MgCl_2_, 1 mM NaF, and 0.02 g sodium azide for 30 min at 37°C. The fraction of enzyme activity inhibited by heparin (1 *μ*g/mL) was considered CK2 activity (this value was evaluated as the difference between the kinase activity measured in the absence of heparin and that measured in the presence of heparin). Whenever indicated, sCK2 activity was measured in the absence or presence of 500 *µ*M putrescine, 500 *µ*M spermidine, and 500 *µ*M spermine. Protein concentration was determined according to the literature using bovine serum albumin (BSA) as a standard [33].

### 2.5. Western Blotting Analyses

CK2 obtained from HPLC purification (1 mg/mL) was submitted to 7.5% SDS-PAGE (sodium dodecyl sulphate-polyacrylamide gel electrophoresis) (26°C/150 V/60 mA), transferred to a PVDF membrane (4°C/100 V/300 mA), and blocked in a buffer containing 150 mM NaCl, 0.05% Tween 20, 5% BSA, and 10 mM Tris (TBS-Tween-BSA), pH 7.6. CK2 was then incubated in TBS-Tween-BSA, pH 7.6, containing primary anti-human *α*CK2 (goat polyclonal IgG, dilution 1 : 7,500). The sample was then washed with TBS-Tween-BSA, pH 7.6, incubated in the same buffer containing secondary antibody (donkey anti-goat IgG conjugated to peroxidase, dilution 1 : 25,000), and analysed using a SuperSignal West Pico kit (Pierce) and Kodak Diagnostic Film (T-Mat S).

### 2.6. Endogenous Phosphorylation Assays

Promastigotes were washed 3 times with PBS and incubated in a buffer containing 150 mM NaCl, 20 mM Tris-HCl, pH 7.5, 10 mM NaF, 1 mM sodium orthovanadate, 150 mM NaCl, 1 mM glucose, and 1 mM MgCl_2_ in the absence (constitutively secreted CK2 [CsCK2]) or presence (inducible secreted activity [IsCK2]) of dephosphorylated casein (1 mg/mL) for 30 minutes. After incubation, the parasites were harvested by centrifugation, and the cell-free supernatant (1 mg/mL) was assayed for endogenous phosphorylation activity [31] in the presence of 100 *µ*M ATP-[*γ*-P32] (1000 cpm/pmol) and a reaction mixture containing 20 mM Tris-HCl, pH 8.0, 150 mM NaCl, 1 mM EDTA, 1 mM EGTA, 1 mM NaF, and 0.02 g sodium azide. Where indicated, the PKC inhibitor bisindolylmaleimide (BIS; 0.4 *µ*M) and the CK2 inhibitors heparin (10 *µ*g/mL) and TBB (1 *µ*M) were used. The reaction was conducted at 37°C for 30 minutes and stopped by increasing the temperature to 100°C for 2 minutes. The samples were analysed by 7.5% SDS-PAGE and stained by Coomassie Brilliant Blue R250. The phosphoproteins were revealed by exposure to Phosphorimager.

### 2.7. Interaction between Murine Macrophages and* L. braziliensis* Parasites

Peritoneal macrophages were extracted from BALB/c and maintained in RPMI 1640 medium at 37°C in a 4% CO_2_ atmosphere for 4 hours. The parasites were harvested by centrifugation, washed twice with 0.9% saline, and then washed once with RPMI 1640 medium. The promastigotes were maintained in contact with the macrophages for 2 hours at a ratio of 10 parasites: 1 macrophage. Then, the supernatant with the unbound parasites was removed. The macrophages were washed with saline solution and incubated for 2 more hours. The macrophages were then fixed, stained with Giemsa, and observed by optical microscopy. Virulent parasites or macrophages were preincubated in the absence or presence of DMSO (1%, drugs diluents), TBB (1 *µ*M, CK2 inhibitor), DRB (6 *µ*M, CK2 inhibitor), putrescine, spermine, and spermidine (500 *µ*M, CK2 stimulators). These drugs were also added to the medium during the interaction experiment. The supernatant of the incubation medium of* L. braziliensis* virulent parasites (1 mg/mL) and the secreted CK2 purified from this supernatant (1 mg/mL) were added to the medium during the interaction experiment between avirulent promastigotes and macrophages or between latex beads and macrophages in the absence or presence of TBB (1 *µ*M). Latex beads (100 nm) were used in the macrophage interaction assay at a dilution 1 : 20. The assay conditions were the same used for the promastigote-macrophage interaction assay.

### 2.8. Statistical Analysis

All results are presented as the mean and standard error of the mean (SEM). Normalised data were analysed with one-way analysis of variance (ANOVA), and differences between groups were assessed by Tukey's post hoc test. A *P* value of <0.05 was considered significant. All the experiments were performed in triplicate in 3 independent experimental sets.

## 3. Results

Virulent and avirulent parasites exhibited differences in protein kinase activity in the supernatant of promastigotes incubated in a specific buffer (Table 1). The virulent live promastigotes presented high specific activity for CK2 (34.400 ± 1.340 pmol Pi·mg/min) compared with the avirulent strain (0.040 ± 0.001 pmol·Pi mg^−1^·min^−1^). The sCK2 activity of the virulent sample was increased (91.5%) compared with the previous incubation of intact cells with extracellular dephosphorylated casein, indicating that the total enzyme levels secreted to the medium could be enhanced by the previous presence of a classical* in vitro* CK2 substrate, such as casein. Thus, from this point, we will make reference to general sCK2 activity, and whenever necessary, the different pools of the enzyme obtained by constitutive secretion will be referred to as CsCK2. The increase in enzyme activity exhibited by substrate induction will be referred to as IsCK2 (Table 1). In the virulent sample, at least 62% of the secreted kinase activities or 53% of ectokinase activity measured in the conditions described here corresponded to CK2 activity. In the avirulent sample, this value was 50% (data not shown). The secreted kinase activity of the virulent sample was arbitrarily considered to be 100% of the nonspecific kinase activity when using dephosphorylated casein as substrate, and the activity sensitive to heparin (1 *μ*g/mL) was considered CK2 activity (Table 2). Based on the secreted kinase activity using the CK2-specific peptide, the actual percentage of specifically CK2 activity was estimated by comparison with that obtained using casein. Thus, under the conditions used in the present study, 79.51% of the secreted kinase activity was attributed specifically to CK2. However, the difference between kinases activities measured using dephosphorylated casein and CK2-specific peptide as a substrate was not significant. The activity measured using the CK2 peptide as a substrate was abolished by heparin (inhibition of 94.35%). The viability of* L. braziliensis *promastigotes did not significantly change during the course of all the experiments presented in this study, as assessed by the Trypan blue dye exclusion method.

The CK2 activities assayed in the virulent strain were at least 121-fold more pronounced than the activities exhibited by the avirulent sample. Dephosphorylated casein stimulated sCK2 in approximately 92% of the virulent parasites and did not affect the avirulent parasites (Table 1).

**Figure 1:**
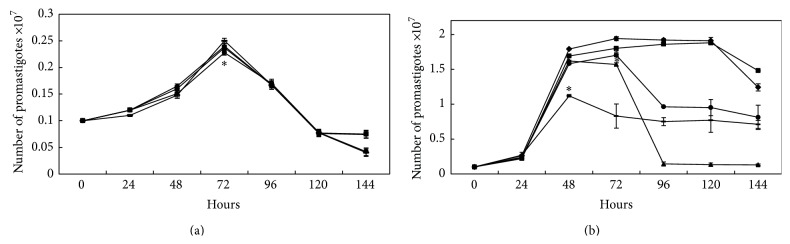
Effect of specific CK2 inhibitors (heparin, TBB, and DRB) on the proliferationof* L. braziliensis *avirulent (a) and virulent (b) samples. The assay was performed as described in Section 2. The values are presented as the mean of 3 independent experiments conducted in triplicate. In (a), (^*^) heparin and DRB significantly inhibited proliferation (*P* ≤ 0.05) after 72 hours. In (b), (^*^) heparin significantly inhibited proliferation (*P* ≤ 0.002) after 48 hours, and TBB and DRB significantly inhibited proliferation (*P* ≤ 0.01) after 72 hours (one-way ANOVA and Tukey's post hoc test) (◆ Control; ■ 1 mM Casein; ▲ 1 *µ*M TBB; — 10 *µ*g/mL Heparin; ● 6 *µ*M DRB).

We tested the ability of traditional CK2 inhibitors, TBB, DRB, and heparin, to interfere with parasite growth [20, 26]. These inhibitors had no effect on the growth of avirulent promastigotes (Figure 1(a)). However, such inhibitors triggered significantly decreased virulent promastigote growth (Figure 1(b)). TBB was the most effective inhibitor and completely abolished parasite growth after 96 h (Figure 1(b)). Heparin induced 37% inhibition of cell growth at an earlier phase (48 h) compared with the other inhibitors (Figure 1).

**Figure 2:**
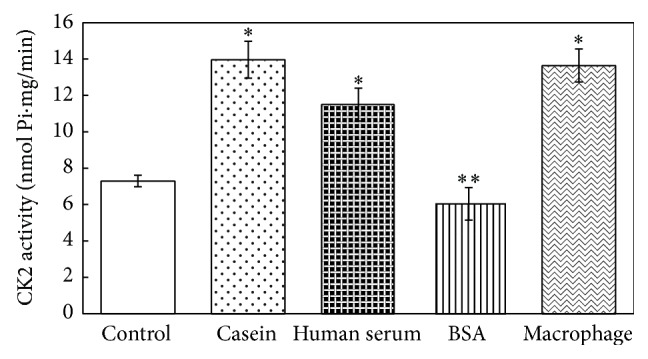
Effect of possible substrates on sCK2 activity in the virulent sample of* L. braziliensis*. CK2 activity was measured as previously described [32], and the conditions of the assay are described in Section 2 (all substrates were used at 1 mg/mL). The values are presented as the mean of 3 independent experiments conducted in triplicate (∗ indicates *P* ≤ 0.002 and ∗∗ indicates *P* ≤ 0.01; one-way ANOVA and Tukey's post hoc test).

Spermine, a polyamine that activates CK2, induced the greatest effect on these activities, whereas constitutive secreted CK2 activity exhibited a maximum activation of 247% (Table 3). The smallest effect was caused by putrescine, whereas the greatest stimulation occurred with the IsCK2 (54%; Table 3). Inactivated human serum and fixed macrophages increased secreted CK2 activity by 58% and 87%, respectively (Figure 2). BSA did not interfere with this activity (Figure 2).

**Figure 3:**
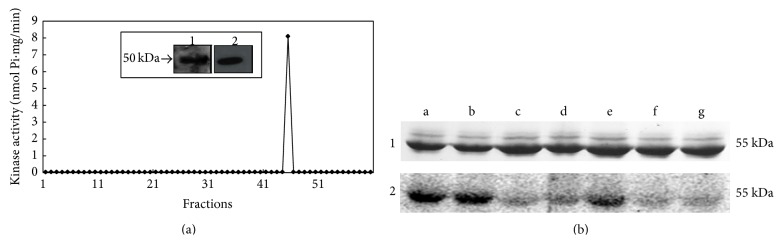
Partial purification of the sCK2 in the virulent sample of* L. braziliensis*. (a) Identification of kinase activity. All of the obtained fractions were assayed for CK2 activity as described in Section 2 [32].* Inset*. Western blotting of the purified secreted CK2 of the virulent* L. braziliensis *sample. Fraction 45 was submitted to 12% SDS-PAGE (Lane 1), transferred to a PVDF membrane (Lane 2), incubated with an anti-*α*CK2 antibody, and developed as described in Section 2. (b) Phosphorylation of the supernatant proteins of the virulent* Leishmania braziliensis* sample by the CK2 enzyme secreted by this parasite. After the supernatant was obtained in the absence (control) or presence (experimental) of dephosphorylated casein, the assay was performed as described in Section 2. (1) Proteins stained with Coomassie Brilliant Blue R250. (2) Phosphorylated proteins observed by exposure to a phosphorimager plate (a: control supernatant, b: experimental supernatant, c: control supernatant + TBB, d: experimental supernatant + TBB, e: control supernatant + BIS, f: control supernatant + heparin, and g: experimental supernatant + heparin).

The parasite supernatant, which was obtained after incubation with 1 mg/mL dephosphorylated casein for 30 min, was fractionated by gel filtration chromatography. The samples recovered from the HPLC column were assayed for CK2 activity as described in Materials and Methods. Only one fraction (fraction 45) presented kinase activity (Figure 3(a)). This kinase activity (9.790 ± 0.040 pmol·Pi mg^−1^·min^−1^) was abolished by TBB and heparin (data not shown). This fraction presented a 50 KDa protein (Figure 3(a),* Inset*, Lane 1), which was recognised by polyclonal antibodies against the human *α*-CK2 subunit (Figure 3(a),* Inset*, Lane 2).

The supernatant obtained from virulent promastigotes incubated in the absence (Figure 3(b), Lanes a, c, e, and f) or presence (Figure 3(b), Lanes b, d, and g) of dephosphorylated casein was phosphorylated* in vitro* and presented a major band with a molecular mass of 55 kDa (Figure 3(b), Line 1). This protein was phosphorylated by the secreted contents of virulent promastigotes (Figure 3(b), Line 2). TBB (Figure 3(b), Line 2, Lanes c and d) and heparin (Figure 3(b), Line 2, Lanes f and g) significantly decreased this phosphorylation. The PKC inhibitor bisindolylmaleimide (Figure 3(b), Line 2, Lane e) did not cause alterations in the phosphorylation profile.

**Figure 4:**
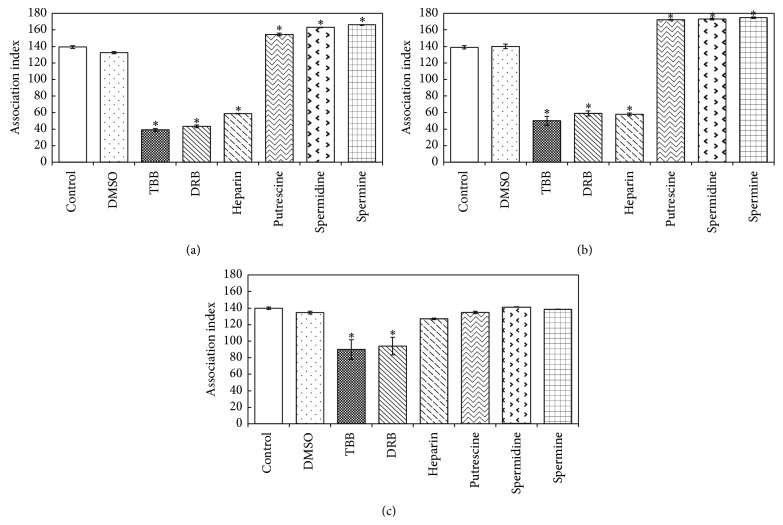
Effect of CK2 inhibitors on virulent* L. braziliensis* sample and macrophages. The assay was performed as described in Section 2. (a) Inhibitors added during the interaction experiment. (b) Promastigotes pretreated with inhibitors. (c) Macrophages pretreated with inhibitors. The values represent the mean of 3 independent experiments conducted in duplicate (∗ indicates *P* ≤ 0.0001 and ∗∗ indicates *P* ≤ 0.003; one-way ANOVA and Tukey's post hoc test).

TBB, DRB, and heparin were used as tools to determine the role of CK2 in the host-parasite interaction. Therefore, these inhibitors were added during a cell interaction assay (Figure 4(a)), and either the parasite (Figure 4(b)) or the macrophage (Figure 4(c)) was treated with the inhibitors before the cell interaction assay. All of the inhibitors decreased the association index between virulent promastigotes and macrophages. DMSO, a diluent of drugs, had no effect on this interaction process (Figure 4). The inhibition effect was more pronounced when the inhibitors were added during the interaction experiments. TBB, DRB, and heparin inhibited the interaction process to 72%, 69%, and 58% of control levels, respectively (Figure 4(a)). Similarly, the inhibition was stronger when the parasites were pretreated with these drugs, as the interaction was inhibited to approximately 60% of control levels (Figure 4(b)). In contrast, the pretreatment of macrophages with TBB and DRB caused a weak inhibition (~34%), whereas heparin had no effect (Figure 4(c)), likely due to drug distribution to the intracellular milieu during the previous period of cell incubation with the drugs. Thus, such drugs must still be available in the extracellular environment where sCK2 is secreted. The polyamines putrescine, spermidine, and spermine increased the association index of virulent promastigotes and macrophages by 23% (Figures 4(a) and 4(b)) but had no effect when only the macrophages were pretreated (Figure 4(c)).

**Figure 5:**
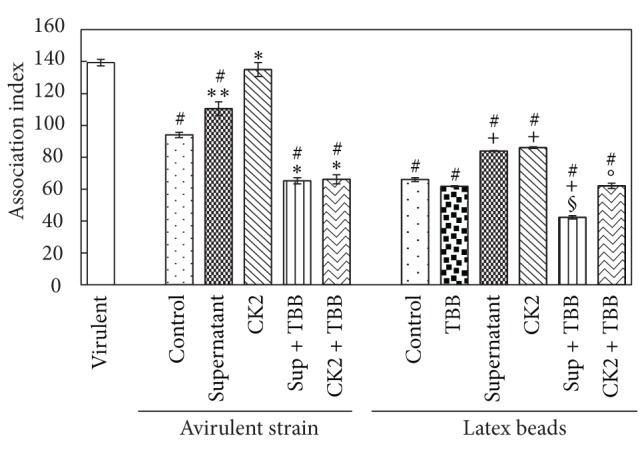
Effect of purified CK2 and the supernatant of the virulent strain on the interaction between the* L. braziliensis* avirulent strain and macrophages and the phagocytosis of latex beads. The assay was performed as described in Section 2. The values are presented as the mean of 3 independent experiments conducted in duplicate (∗ indicates *P* ≤ 0.001 and ∗∗ indicates *P* ≤ 0.03 in relation to control avirulent strain; # indicates *P* ≤ 0.003 in relation to virulent strain; + indicates *P* ≤ 0.005 in relation to control latex beads; § indicates *P* ≤ 0.05 in relation to supernatant of latex beads; and ° indicates *P* ≤ 0.05 in relation to CK2 of latex beads; one-way ANOVA and Tukey's post hoc test).

The virulent strain showed an association index with host cells approximately 48% higher than that of the avirulent strain (Figures 4 and 5, Table 4). The effect of CK2 modulators in the interaction process between avirulent promastigotes and macrophages was less pronounced (Table 4). Heparin promoted an inhibition of 20% in the pretreated macrophages, 31% when the parasites and macrophages were treated together, and 46% in the pretreated parasites (Table 4). In contrast, TBB promoted an inhibition of only 16% when the parasites and macrophages were treated together and 22% in the pretreated parasites. This inhibitor had no effect when the macrophages were pretreated (Table 4). Spermine promoted a maximum enhancement of only 19% in the pretreated parasites. On the other hand, it caused an inhibition of 17% when the macrophages were pretreated (Table 4).

The supernatant of virulent promastigotes and the purified CK2 fraction (fraction 45) were tested for their ability to promote macrophage invasion. These sources of CK2 caused an enhancement of 18% and 44%, respectively, in the association index between avirulent promastigotes and macrophages. This effect was abolished by TBB (Figure 5). The same effect could be observed on the phagocytosis of latex beads. This enzyme promoted a great enhancement in this process that was totally inhibited by TBB (Figure 5), showing that the effect also occurred in the macrophage.

## 4. Discussion

Posttranslational modifications greatly impact the activity of certain proteins that play key roles in several cellular processes ranging from metabolism to cell growth, proliferation, and differentiation. The reversible phosphorylation of proteins mediated by protein kinases and phosphatases is crucial for the intracellular signal transduction pathways involved in these processes. Several protein kinases [23–30] and phosphatases [34–41] have been identified in trypanosomatids and are likely involved in the regulation of the cell cycle, cell differentiation, and responses to stress during their complex life cycles. These enzymes have frequently been associated with the parasite-host relationship [23–25, 27, 35, 36, 42]. The presence of these enzymes in these parasites was confirmed by sequencing the* T. cruzi, T. brucei, *and* L. major *genomes. These sequencing efforts made the kinome prediction of these parasites possible. Kinome analysis has revealed that the trypanosomatids lack members of the receptor-linked or cytosolic tyrosine kinase families but have an abundance of soluble protein kinases [43]. The presence of CK2 activity has been previously described in trypanosomatids [23–27]. Kinome analysis of* L. major* has revealed the presence of six isoforms of CK1 and two isoforms of CK2 in this species. In addition, this last enzyme was shown to be distributed on the external surface of the parasite (ecto-CK2 activity), in the cytoplasmic content (intracellular CK2 activity), and in the secreted content (secreted CK2 activity) by this parasite. These enzyme activities phosphorylated either casein or phosvitin. However, the amino acid sequences of these enzymes are different from their mammalian homologues [25, 27, 43]. Accordingly, we show here that considering the phosphorylation of casein by the supernatant of live* L. braziliensis* virulent promastigotes, 53% of the ectokinase and 62% of the secreted kinase activities were attributed to CK2. The remaining ectokinases activities (47%) and secreted kinase activities (38%) corresponded to casein kinase activities of unknown identity. These activities could be due to other isoforms of CK1 and CK2 or even other protein kinases. Heparin abolished the phosphorylation of casein and the phosphorylation of a substrate peptide by the CK2 secreted enzyme. Heparin has been used to identify CK2 activity because its IC_50_ for CK1 is 160-fold higher than that for CK2 [44].

*L. braziliensis* virulent promastigotes exhibited significantly higher activity (at least 121-fold) than the avirulent promastigotes. These protein kinases appear to be important for the growth of virulent parasites because their inhibition resulted in inhibited cell growth. In addition, CK2 inhibitors (heparin, DRB, and TBB) strongly inhibited the growth of the virulent strain but had no effect on the avirulent strain. Similar to heparin, TBB and DRB are specific CK2 inhibitors [8]. The effects of heparin and TBB were more pronounced than DRB, and TBB abolished protozoan growth. Therefore, CK2 activity appears to be extremely important for the survival of the* L. braziliensis *virulent strain, which has also been shown for CK2 activity in* L. chagasi* [45], implying an association between enzyme activity and parasite infectivity. Similarly, this enzyme is important for another parasite,* Plasmodium falciparum*, as gene disruption experiments have shown that CK2 is essential for blood schizogony [46].

This first set of results indicates that these parasites possess a significant array of CK2 enzymes expressed in different cell compartments and that they differ in their ability to respond to extracellular protein targets. In addition, such differences were positively correlated with the infectivity of the parasite and prompted us to investigate the general aspects of CK2 biochemistry in these cells.

Similarly, the induced CK2 secretion of* L. braziliensis* virulent promastigotes was strongly stimulated by incubation with this substrate. In* L. major*, the secreted CK1 activity is constitutive [25], whereas the release of CK2 is only stimulated by the incubation of the parasite with the substrate.* L. major* promastigotes release both constitutive and inducible protein kinases with different activities [25], suggesting that ecto- and secreted protein kinases may play an important role in parasite survival. In* L. donovani*, both enzymes are present constitutively [27].* L. tropica *secretes CK2 constitutively, whereas the secreted CK2 activity is induced by incubation with dephosphorylated casein [23].

The secretion of CK2 may play a vital role in parasite survival inside the vertebrate host cells, that is, macrophages. Phosphorylation of host serum or host cell proteins may be involved in the regulation of leishmanicidal processes. The components of these substances can be used as substrates by the CK2 activity of the parasite. The presence of inactivated human serum and murine macrophages enhances the secreted CK2 activity of the virulent strain. This stimulation was equivalent to that induced by casein. Foetal calf serum and bovine serum albumin did not influence CK2 activity. The macrophages and human serum may present substrates for secreted CK2 from* L. braziliensis*, whereas exogenous substrates can promote CK2 release [25, 27].

To further investigate the nature of the enzymes responsible for secreted* L. braziliensis *kinase activity, we demonstrated that polyclonal antibodies raised against the mammalian CK2*α* catalytic subunit recognised a protein secreted by* L. braziliensis *promastigotes. These promastigotes were incubated in the same incubation buffer used for the secreted kinase assays and were partially isolated by HPLC chromatography. Approximately 60 fractions were harvested with this method. Only one fraction (fraction 45) presented kinase activity, which was readily abolished by heparin and TBB, confirming the possibility that this activity was from CK2. This assay showed a single band with a molecular weight (50 kDa) compatible with the subunit of the CK2 enzyme of other trypanosomatids, such as* H. m. muscarum* [26] and* L. tropica *[23].

The secreted CK2 activity of the virulent sample of* L. braziliensis* phosphorylated a protein secreted by this same parasite. This protein weighs approximately 55 kDa, and phosphorylation was strongly inhibited by TBB and heparin, specific CK2 inhibitors, whereas no effect was observed for the PKC inhibitor bisindolylmaleimide. The phosphorylation of macrophage receptors during the* Leishmania* interaction and phagocytosis can act synergistically with other virulence factors, such as lipophosphoglycan (LPG). This major surface glycoconjugate of* Leishmania* is transferred from the parasite to the host macrophage membrane during phagocytosis and seems to promote blockage of macrophage activation, protecting the parasite [47]. The autophosphorylation of some protein kinases, such as PKC, has been observed in mammal cells [48] and in* T. cruzi* [49]. In addition to autophosphorylation, the phosphorylation of other molecules present on the parasite, such as secreted proteins, can also be part of the complex signalling pathway of this protozoan.

Polyamines such as putrescine, spermidine, and spermine are positively charged molecules and are thus able to increase constitutive and inducible virulent ecto- and secreted CK2 activity. Spermine exhibited the most pronounced effect on constitutive secreted CK2 activity. This effect is likely due to the four positive charges of this molecule [50]. Moreover, its structure is the most compatible with the CK2 structure because the stability of *α* and *β* binding is due to four electrostatic interactions [16].

The possible role of ecto- or secreted enzymes in parasite-host interactions has been suggested by several authors [23, 24, 27, 34–36, 42]. The CK2 activity of* L. major *and* L. amazonensis* influences the cell growth, morphology, and infectivity of the promastigotes in murine macrophages* in vitro* and BALB/c mice* in vivo* [24]. Two of the CK2 inhibitors, TBB and DRB, strongly decreased the association index between the* L. braziliensis* virulent sample and murine macrophages when added during the interaction assay, whereas heparin exhibited a minor effect. When these drugs were used to pretreat the parasites or macrophages, some differences were observed. The CK2 inhibitors were more potent in inhibiting the interaction when the parasites were pretreated than when the macrophages were pretreated; however, heparin did not inhibit this process. The polyamines increased the virulent sample index but had no effect when the macrophages were pretreated. These data suggest that the parasite enzymes may play a more important role in this process than the host cell enzymes.

As expected, the association index of the virulent sample was 48% higher than that of the avirulent sample; therefore, the CK2 modulators had a less pronounced effect. Interestingly, the avirulent sample association index increased with the addition of supernatant from the virulent strain or purified CK2. The CK2 isolated from the parasite showed a more potent effect because it increased the association index to the same value as that found in the virulent strain. TBB inhibited the purified CK2 and supernatant effects. The same effect was observed in the association index between* L. tropica* and macrophages with CK2 purified from another source, which enhanced the index by 166%. Heparin, a specific CK2 inhibitor, inhibited the association index by 50% [23]. sCK2 significantly affects the phagocytosis of latex beads by macrophages, indicating that this enzyme interferes with these cells. These results, together with the fact that macrophage extract increased sCK2 activity, suggest that the effect on the macrophage most likely occurs due to phosphorylation of some substrates capable of interfering with the phagocytosis process. These data demonstrate the importance of CK2 activity on the* Leishmania*-host interaction.

The survival of* Leishmania* inside macrophages depends on two inducible enzymes: iNOS and arginase. Both of these enzymes use L-arginine as substrate; iNOS (inducible NO sintase) produces NO (nitric oxide) (the most important leishmanicidal molecule) from L-arginine through two steps, whereas arginase catalyzes the hydrolysis of L-arginine into ornithine, the primary source of polyamines [51]. The* Leishmania* infection can induce host IL-4 production [52], which changes macrophage L-arginine metabolism to polyamine production by arginase activation [51]. These molecules promote parasite growth [51], interfere with signalling pathways, and act as immunosuppressors [53]. Furthermore, these molecules act as activators of leishmanial CK2 activities, promoting parasite-host cell interactions, as observed in the present study (Figure 4).

The maintenance of the virulent status of these parasites and their interaction with the host are essential for their life cycle. These processes are very complex and involve several signal transduction pathways. The study of factors that modulate the* Leishmania*-host interaction and the signalling routes of these processes may be of the great value for controlling the diseases associated with this parasite. The knowledge generated by these studies can be used in the development of novel, potentially less toxic, and more effective drugs than those currently used to treat leishmaniasis. Similarly, this knowledge may ultimately lead to new preventive strategies that target specific parasite molecules involved in the regulation of parasite survival and the infectivity of host cells.

## Figures and Tables

**Table 1:** Effect of casein on the secreted CK2 activities of *Leishmania braziliensis* avirulent and virulent samples.

Systems	Virulent strain	Avirulent strain	Virulent activity/avirulent activity
	pmol·Pi mg^−1^·min^−1^ ± E.P.	pmol·Pi mg^−1^·min^−1^ ± E.P.	
Constitutive ecto-CK2 activity (eCK2)	34.400 ± 1.340	0.040 ± 0.001	860.00
Constitutive intracellular CK2 activity (iCK2)	4.800 ± 0.610	0.290 ± 0.002	16.55
Constitutive secreted CK2 activity (CsCK2)	7.290 ± 0.320	0.060 ± 0.006	121.50
Inducible secreted CK2 activity (IsCK2)	13.960 ± 1.010^*^	0.070 ± 0.006	199.43

The reactions were performed as described in Section 2. The values represent the mean of 3 independent experiments conducted in triplicate (∗ indicates *P* ≤ 0.005 in relation to CsCK2; one-way ANOVA, Tukey's post hoc test).

**Table 2:** Secreted kinase activity of *Leishmania braziliensis *promastigotes using different substrates.

Substrates	Secreted kinase activity (pmol·Pi mg^−1^·min^−1^)	Percentage (%)^a^	Percentage (%)^b^
Casein	7.282 ± 0.476	100	—
CK2-specific Peptide	5.790 ± 0.245	79.510	100
CK2-specific peptide + heparin	0.327 ± 0.210^*^	4.490	5.648

The reactions were performed as described in Section 2. The values are presented as the mean ± SEM of at least 3 independent experiments, which were performed in triplicate (^*^ *P* < 0.05 in relation to CK2-specific peptide; one-way ANOVA, Tukey's post hoc test).

^a^The kinase activity was arbitrarily considered to be 100% when the value was obtained using casein as substrate. Based on the secreted kinase activity using CK2-specific peptides, the percentage was estimated by comparison with that obtained using casein, whereas the activity sensitive to heparin (1 *μ*g/mL) was considered CK2 activity.

^b^The kinase activity was arbitrarily considered to be 100% when the value was obtained using the CK2 peptide as substrate.

**Table 3:** Effect of polyamines on the constitutive and inducible secreted CK2 activity of the *Leishmania braziliensis* virulent strain.

Addition	Constitutive secreted CK2		Inducible secreted CK2	
	nmol Pi/mg·min^−1^ ±SE	% kinase activity	nmol Pi/mg·min^−1^ ±SE	% kinase activity
None	7.70 ± 0.63	100.00	13.96 ± 0.64	100.00
Putrescine (500 *μ*M)	14.48 ± 0.52^**^	188.01	21.50 ± 1.15^**^	154.01
Spermidine (500 mM)	18.00 ± 0.84^**^	233.77	25.32 ± 0.24^*^	181.38
Spermine (500 mM)	26.75 ± 0.72^*^	347.40	32.76 ± 0.26^*^	234.67

The reactions were performed as described in Section 2. The values are presented as the mean of 3 independent experiments conducted in triplicate (∗ indicates *P* ≤ 0.0002, ∗∗ indicates *P* ≤ 0.007, and ∗∗∗ indicates *P* ≤ 0.05 compared with control without addition; one-way ANOVA, Tukey's post hoc test).

**Table 4:** Effect of CK2 modulators on the interaction between the *L. braziliensis* avirulent strain and macrophages.

Addition	Addition during interaction course		Avirulent pretreated parasites		Pretreated macrophages	
	Association index ± SE	% interaction	Association index ± SE	% interaction	Association index ± SE	% interaction
None	91.69 ± 2.08	100.00	91.78 ± 2.11	100.00	91.68 ± 1.64	100.00
TBB (1 *μ*M)	76.89 ± 1.27^**^	83.86	71.83 ± 1.94^*^	78.26	83.96 ± 1.92	91.58
Heparin (10 *μ*g/mL)	62.99 ± 0.80^*^	68.70	53.56 ± 0.91^*^	54.77	73.28 ± 1.34^*^	79.93
Spermine (500 mM)	97.43 ± 1.11	106.26	109.25 ± 0.72^*^	119.03	76.35 ± 0.95^*^	83.28

The reactions were performed as described in Section 2. The values are presented as the mean of 3 independent experiments conducted in triplicate (∗ indicates *P* ≤ 0.009 and ∗∗ indicates *P* ≤ 0.05 compared with control without addition; one-way ANOVA, Tukey's post hoc test).

## References

[1] Soares R. P., Margonari C., Secundino N. C. (2010). Differential midgut attachment of *Leishmania* (*Viannia*) *braziliensis* in the sand flies *Lutzomyia* (*Nyssomyia*) *whitmani* and *Lutzomyia* (*Nyssomyia*) *intermedia*. *Journal of Biomedicine and Biotechnology*.

[2] Alexander J., Russell D. G. (1992). The interaction of *Leishmania* species with macrophages. *Advances in Parasitology*.

[3] Parsons M., Ruben L. (2000). Pathways involved in environmental sensing in trypanosomatids. *Parasitology Today*.

[4] Lopes A. H., Souto-Padrón T., Dias F. A. (2010). Trypanosomatids: odd organisms, devastating diseases. *The Open Parasitology Journal*.

[5] Manning G., Plowman G. D., Hunter T., Sudarsanam S. (2002). Evolution of protein kinase signaling from yeast to man. *Trends in Biochemical Sciences*.

[6] Pinna L. A. (1990). Casein kinase 2: an “eminence grise” in cellular regulation?. *Biochimica et Biophysica Acta*.

[7] Blanquet P. R. (2000). Casein kinase 2 as a potentially important enzyme in the nervous system. *Progress in Neurobiology*.

[8] Litchfield D. W. (2003). Protein kinase CK2: structure, regulation and role in cellular decisions of life and death. *Biochemical Journal*.

[9] Allende J. E., Allende C. C. (1995). Protein kinase CK2: an enzyme with multiple substrates and a puzzling regulation. *FASEB Journal*.

[10] Lozeman F. J., Litchfield D. W., Piening C., Takio K., Walsh K. A., Krebs E. G. (1990). Isolation and characterization of human cDNA clones encoding the *α* and the *α*′ subunits of casein kinase II. *Biochemistry*.

[11] Litchfield D. W., Lozeman F. J., Piening C. (1990). Subunit structure of casein kinase II from bovine testis. Demonstration that the *α* and *α*′ subunits are distinct polypeptides. *The Journal of Biological Chemistry*.

[12] Pinna L. A. (2002). Protein kinase CK2: a challenge to canons. *Journal of Cell Science*.

[13] Litchfield D. W., Bosc D. G., Canton D. A., Saulnier R. B., Vilk G., Zhang C. (2001). Functional specialization of CK2 isoforms and characterization of isoform-specific binding partners. *Molecular and Cellular Biochemistry*.

[14] Shi X., Potvin B., Huang T. (2001). A novel casein kinase 2 *α*-subunit regulates membrane protein traffic in the human hepatoma cell line HuH-7. *The Journal of Biological Chemistry*.

[15] Filhol O., Martiel J.-L., Cochet C. (2004). Protein kinase CK2: a new view of an old molecular complex. *EMBO Reports*.

[16] Leroy D., Heriché J.-K., Filhol O., Chambaz E. M., Cochet C. (1997). Binding of polyamines to an autonomous domain of the regulatory subunit of protein kinase CK2 induces a conformational change in the holoenzyme a proposed role for the kinase stimulation. *The Journal of Biological Chemistry*.

[17] Meggio F., Pinna L. A. (2003). One-thousand-and-one substrates of protein kinase CK2?. *The FASEB Journal*.

[18] Singh N. N., Ramji D. P. (2008). Protein kinase CK2, an important regulator of the inflammatory response?. *Journal of Molecular Medicine*.

[19] Zandomeni R., Zandomeni M. C., Shugar D., Weinmann R. (1986). Casein kinase type II is involved in the inhibition by 5,6-dichloro-1-*β*-D-ribofuranosylbenzimidazole of specific RNA polymerase II transcription. *The Journal of Biological Chemistry*.

[20] Mesquita R. D., de Oliveira F. M. B., Shugar D., Fantappié M. R., Silva-Neto M. A. C. (2005). Nitrophorin synthesis is modulated by protein kinase CK2. *Biochemical and Biophysical Research Communications*.

[21] Kübler D., Pyerin W., Kinzel V. (1982). Protein kinase activity and substrates at the surface of intact HeLa cells. *The Journal of Biological Chemistry*.

[22] Kübler D., Pyerin W., Burow E., Kinzel V. (1983). Substrate-effected release of surface-located protein kinase from intact cells. *Proceedings of the National Academy of Sciences of the United States of America*.

[23] Dutra P. M. L., Vieira D. P., Meyer-Fernandes J. R., Silva-Neto M. A. C., Lopes A. H. (2009). Stimulation of *Leishmania tropica* protein kinase CK2 activities by platelet-activating factor (PAF). *Acta Tropica*.

[24] Becker S., Jaffe C. L. (1997). Effect of protein kinase inhibitors on the growth, morphology, and infectivity of *Leishmania* promastigotes. *Parasitology Research*.

[25] Sacerdoti-Sierra N., Jaffe C. L. (1997). Release of ecto-protein kinases by the protozoan parasite *Leishmania major*. *The Journal of Biological Chemistry*.

[26] Silva-Neto M. A. C., Carneiro A. B., Vieira D. P., Mesquita R. D., Lopes A. H. C. S. (2002). Platelet-activating factor (PAF) activates casein kinase 2 in the protozoan parasite Herpetomonas muscarum muscarum. *Biochemical and Biophysical Research Communications*.

[27] Vieira L. L., Sacerdoti-Sierra N., Jaffe C. L. (2002). Effect of pH and temperature on protein kinase release by *Leishmania donovani*. *International Journal for Parasitology*.

[28] Calabokis M., Kurz L., Wilkesman J. (2002). Biochemical and enzymatic characterization of a partially purified casein kinase-1 like activity from *Trypanosoma cruzi*. *Parasitology International*.

[29] Park J.-H., Brekken D. L., Randall A. C., Parsons M. (2002). Molecular cloning of *Trypanosoma brucei* CK2 catalytic subunits: the *α* isoform is nucleolar and phosphorylates the nucleolar protein Nopp44/46. *Molecular and Biochemical Parasitology*.

[30] Donald R. G. K., Zhong T., Meijer L., Liberator P. A. (2005). Characterization of two *T. gondii* CK1 isoforms. *Molecular and Biochemical Parasitology*.

[31] Barankiewicz J., Dosch H.-M., Cohen A. (1988). Extracellular nucleotide catabolism in human B and T lymphocytes. The source of adenosine production. *The Journal of Biological Chemistry*.

[32] Silva-Neto M. A. C., Oliveira P. L. (1993). Protein phosphorylation in *Rhodnius prolixus* oocytes: identification of a type II casein kinase. *Insect Biochemistry and Molecular Biology*.

[33] Lowry O. H., Rosebrough N. J., Farr A. L., Randall R. J. (1951). Protein measurement with the Folin phenol reagent. *The Journal of Biological Chemistry*.

[34] Gottlieb M., Dwyer D. M. (1981). Protozoan parasite of humans: surface membrane with externally disposed acid phosphatase. *Science*.

[35] Remaley A. T., Glew R. H., Kuhns D. B. (1985). *Leishmania donovani*: surface membrane acid phosphatase blocks neutrophil oxidative metabolite production. *Experimental Parasitology*.

[36] Vannier-Santos M. A., Martiny A., Meyer-Fernandes J. R., de Souza W. (1995). Leishmanial protein kinase C modulates host cell infection via secreted acid phosphatase. *European Journal of Cell Biology*.

[37] Dutra P. M. L., Rodrigues C. O., Jesus J. B., Lopes A. H. C. S., Souto-Padrón T., Meyer-Fernandes J. R. (1998). A novel ecto-phosphatase activity of *Herpetomonas muscarum muscarum* inhibited by platelet-activating factor. *Biochemical and Biophysical Research Communications*.

[38] Dutra P. M. L., Rodrigues C. O., Romeiro A. (2000). Characterization of ectophosphatase activities in trypanosomatid parasites of plants. *Phytopathology*.

[39] Dutra P. M. L., Dias F. A., Rodrigues C. O. (2001). Platelet-activating factor modulates a secreted phosphatase activity of the trypanosomatid parasite *Herpetomonas muscarum* muscarum. *Current Microbiology*.

[40] Dutra P. M. L., Dias F. A., Santos M. A. A. (2001). Secreted phosphatase activities in trypanosomatid parasites of plants modulated by platelet-activating factor. *Phytopathology*.

[41] Dutra P. M. L., Couto L. C., Lopes A. H. C. S., Meyer-Fernandes J. R. (2006). Characterization of ecto-phosphatase activities of Trypanosoma cruzi: a comparative study between Colombiana and Y strains. *Acta Tropica*.

[42] Martiny A., Vannier-Santos M. A., Borges V. M. (1996). *Leishmania*-induced tyrosine phosphorylation in the host macrophage and its implication to infection. *European Journal of Cell Biology*.

[43] Naula C., Parsons M., Mottram J. C. (2005). Protein kinases as drug targets in trypanosomes and *Leishmania*. *Biochimica et Biophysica Acta*.

[44] Hathaway G. M., Lubben T. H., Traugh J. A. (1980). Inhibition of casein kinase II by heparin. *The Journal of Biological Chemistry*.

[45] Bhatia A., Sanyal R., Paramchuk W., Gedamu L. (1998). Isolation, characterization and disruption of the casein kinase II alpha subunit gene of *Leishmania chagasi*. *Molecular and Biochemical Parasitology*.

[46] Holland Z., Prudent R., Reiser J.-B., Cochet C., Doerig C. (2009). Functional analysis of protein kinase CK2 of the human malaria parasite *Pasmodium falciparum*. *Eukaryotic Cell*.

[47] Winberg M. E., Holm Å., Särndahl E. (2009). *Leishmania donovani* lipophosphoglycan inhibits phagosomal maturation via action on membrane rafts. *Microbes and Infection*.

[48] Huang K.-P., Chan K.-F. J., Singh T. J., Nakabayashi H. (1986). Autophosphorylation of rat brain Ca^2+^-activated and phospholipid-dependent protein kinase. *The Journal of Biological Chemistry*.

[49] Gómez M. L., Ochatt C. M., Kazanietz M. G., Torres H. N., Téllez-Iñón M. T. (1999). Biochemical and immunological studies of protein kinase C from *Trypanosoma cruzi*. *International Journal for Parasitology*.

[50] Korolev N., Lyubartsev A. P., Laaksonen A., Nordenskiöld L. (2004). A molecular dynamics simulation study of polyamine- and sodium-DNA. Interplay between polyamine binding and DNA structure. *European Biophysics Journal*.

[51] Kropf P., Fuentes J. M., Fähnrich E. (2005). Arginase and polyamine synthesis are key factors in the regulation of experimental leishmaniasis in vivo. *The FASEB Journal*.

[52] Himmelrich H., Launois P., Maillard I. (2000). In BALB/c mice, IL-4 production during the initial phase of infection with *Leishmania major* is necessary and sufficient to instruct Th2 cell development resulting in progressive disease. *Journal of Immunology*.

[53] Bogdan C. (2001). Nitric oxide and the immune response. *Nature Immunology*.

